# *biomvRhsmm:* Genomic Segmentation with Hidden Semi-Markov Model

**DOI:** 10.1155/2014/910390

**Published:** 2014-06-03

**Authors:** Yang Du, Eduard Murani, Siriluck Ponsuksili, Klaus Wimmers

**Affiliations:** ^1^Institute for Genome Biology, Leibniz Institute for Farm Animal Biology, 18196 Dummerstorf, Germany; ^2^Research Group Functional Genomics, Leibniz Institute for Farm Animal Biology, 18196 Dummerstorf, Germany

## Abstract

High-throughput technologies like tiling array and next-generation sequencing (NGS) generate continuous homogeneous segments or signal peaks in the genome that represent transcripts and transcript variants (transcript mapping and quantification), regions of deletion and amplification (copy number variation), or regions characterized by particular common features like chromatin state or DNA methylation ratio (epigenetic modifications). However, the volume and output of data produced by these technologies present challenges in analysis. Here, a hidden semi-Markov model (HSMM) is implemented and tailored to handle multiple genomic profile, to better facilitate genome annotation by assisting in the detection of transcripts, regulatory regions, and copy number variation by holistic microarray or NGS. With support for various data distributions, instead of limiting itself to one specific application, the proposed hidden semi-Markov model is designed to allow modeling options to accommodate different types of genomic data and to serve as a general segmentation engine. By incorporating genomic positions into the sojourn distribution of HSMM, with optional prior learning using annotation or previous studies, the modeling output is more biologically sensible. The proposed model has been compared with several other state-of-the-art segmentation models through simulation benchmarking, which shows that our efficient implementation achieves comparable or better sensitivity and specificity in genomic segmentation.

## 1. Introduction

The advent of high-throughput technologies like tiling array and massively parallel sequencing has produced a windfall of large-scale genomic data. Analysis of genome-wide data from these experiments generally requires researchers to search for continuous homogeneous segments or signal peaks. These features can represent regulatory regions [1, 2], transcripts [3–6], or regions of deletion or amplification [7, 8]. The objective of these investigations is, in general, the segmentation or partitioning of the genome into nonoverlapping homogeneous segments and the assignation of a biologically sensible class to each segment.

Various models and computational tools have been developed to handle either the general segmentation problem or particular types of partitioning. Most commonly, the approaches address the detection of chromosomal alterations with array-based comparative genomic hybridization (aCGH) [9–18] or SNP array [19–23], transcript [24, 25] and protein-binding site detection [26, 27] with tiling array, and the identification of gene expression domains [28, 29]. In recent years, more effort has been devoted to the development of computational tools to deal with read-count data generated from next-generation sequencing (NGS) [30–35].

Many of these computational tools utilize hidden Markov model (HMM) [9, 13, 19, 20, 22, 23, 26, 32, 33] because of its inherent capability of resolving segmentation tasks. However, a standard HMM cannot easily account for one basic property of genomic data—the physical position of the feature. To our knowledge, there have been few limited attempts to incorporate this positional information into HMM [13, 22, 23, 26] or to adopt more complex dynamic Bayesian network models [34]. On the other hand, a hidden semi-Markov model (HSMM), a more generalized form of HMM, could be applied to utilize positional information. Indeed, HSMM was proposed for modeling aCGH data [18], but the tool did not actually utilize positional information and the implementation is no longer publicly available.

Here, we implement in the R/Bioconductor [36, 37] package* biomvRCNS* a novel hidden semi-Markov model,* biomvRhsmm*. This model is specially designed to handle genomic data and tailored to serve as a general segmentation tool for various types of genomic profiles arising from both traditional microarray-based experiments and the recent NGS platform, with native support for modeling spatial patterns carried by genomic position. We also compare the proposed model with several other state-of-the-art segmentation models through simulation benchmarking, which shows that our efficient implementation achieves comparable or better sensitivity and specificity in genomic segmentation.

## 2. Materials and Methods

### 2.1. Hidden Semi-Markov Model

A brief summary of the concepts involved and a definition of the hidden semi-Markov model follow. For some experimental data *X*, we have a vector of observations *x*_*t*_ = (*x*_*t*_^1^,…, *x*_*t*_^*N*^) made for *N* samples at each time or position *t*, *t* = 1,…, *T*. At each *t*, there is an underlying unobserved state *S*_*t*_ ∈ *S* = {1,…, *J*}, which depends only on the previous state at *t* − 1, thus forming a length *T* discrete Markov chain with a finite number *J* of possible states. The initial state probability is determined by vector *π*, *π*_*j*_ = *P*(*S*_1_ = *j*), *j* = 1,…, *J*, with ∑_*j*=1_^*J*^ *π*_*j*_ = 1 and *π*_*j*_ ≥ 0. The conditional probability of the observed variable *x*_*t*_ given the unobserved (or hidden) state *j* is referred to as the emission density *B*, *b*_*tj*_ = *P*(*X*_*t*_ = *x*_*t*_ | *S*_*t*_ = *j*). The transition matrix *A*, giving the probabilities of moving from one state to another, is formulated as *a*_*ij*_ = *P*(*S*_*t*+1_ = *j* | *S*_*t*_ = *i*), with ∑_*j*=1_^*J*^ *a*_*ij*_ = 1 and *a*_*ij*_ ≥ 0. Thus an HMM can be defined by *θ* = (*π*, *A*, *B*).

A semi-Markov chain can be considered as a two-layer mixture, an embedded first-order Markov chain representing the transitions between distinct states—which follows the standard definition of HMM—and an occupancy distribution attached to each nonabsorbing state of the embedded first-order Markov chain.

The discrete state occupancy distribution or the sojourn distribution, *D*, is defined as the probability of spending *u* consecutive time steps in state *j*, which is geometrically distributed for a normal HMM, *d*_*j*_(*u*) = *a*_*jj*_^*u*−1^(1 − *a*_*jj*_). The hidden semi-Markov model, with the sojourn distribution explicitly specified using a common distribution, can be defined by *θ* = (*π*, *A*, *B*, *D*). The explicit modeling of sojourn time immediately enables the full inclusion of genomic distance in the segmentation process. A complete likelihood (1) of the HSMM is given in [38] with survivor function *D*_*i*_(*u*) = ∑_*v*≥*u*_ *d*_*i*_(*v*) representing the sojourn time spent in the last state.

Consider

(1) L(θ)=πS1dS1(u1){∏r=2RP(Sr ∣ Sr−1)dSr(ur)} ×P(SR ∣ SR−1)DSR(uR)∏t−1TP(Xt ∣ St).

With likelihood function defined, the optimal model parameters could then be estimated using the expectation-maximization (EM) algorithm. A forward-backward algorithm for the estimation step and a Viterbi algorithm to derive the most likely state sequence are explained in [38], where the author also shows the possibility of replacing the nonparametric M-step of the EM algorithm in sojourn distribution parameters reestimation with a parametric M-step in practice, to simplify the model and prevent overfitting. The E-step of the forward-backward EM procedure and the Viterbi algorithm have been implemented as C library in the package. We also attempted to provide support for parametric reestimations of the M-step based on continuous and discrete distributions like Gamma, Poisson, and negative binomial distribution, which is done* ad hoc* using point estimation methods. More detail about the estimation of HSMM can be found in the Supplementary Material available online at http://dx.doi.org/10.1155/2014/910390.

### 2.2. Implementation

The batch function* biomvRhsmm* accepts both the basic R data matrix and the more encapsulated* GenomicRanges*-like object as input, for better interfacing with other Bioconductor classes and methods [39]. The function will sequentially process each region identified by the distinctive sequence names in the positional input. A second layer of stratification is introduced by a grouping argument, assigning each profile to a group, which could be used to reflect experimental design. Sample columns within the same group could be treated simultaneously in the modeling process as well as iteratively. The assumption is that profiles from the same group could be considered homogeneous and, thus, processed together in a multivariate fashion. Simultaneous treatment of multiple profiles is currently available for emission type set to multivariate normal distribution or multivariate *t* distribution. Additionally, there is a built-in automatic grouping method by hierarchical clustering.

The prior distribution of the sojourn density will be initialized as flat or be estimated from another related data source by calling the function* sojournAnno*. State number could be either assigned explicitly or inferred during the sojourn learning. The model complexity is limited by a constant *M*, denoting the upper bound to the time spent in a state, which is quite similar to the approach adapted in the segmentation model in* tilingArray* [24]. The constant could be explicitly given by the argument max⁡*k* or inferred by another constant max⁡*bp* together with positional information. The modeling of sojourn time is done using positional information like genomic distance between markers and regresses to a rank-based position setting, like the original design in [38], when positional information is not available. Starting state probabilities will be initialized as a flat vector. Initial parameters for the emission distribution could be estimated using different levels of quantile of the input or via a clustering process, assuming different states tend to have different levels of emitted signals.

The function will then call the C library to compute the smoothed-state probability profile in the E-step, after which model parameters will be reestimated in an M-step. Eventually, the most likely state sequence could be inferred from the smoothed-state probability profile or estimated with the Viterbi algorithm. The complexity of the forward-backward algorithm used in the E-step and the Viterbi algorithm is *O*(*JT*(*J* + *T*)) time in the worst case and *O*(*JT* *M*) space. To relax the high memory burden from NGS data of base-pair resolution, we attempt to use run-length encoding (RLE) for the storage and handling of sequencing count data, since the feature distribution is normally sparse across the genome. Also, to speed up computation, parallel processing of multiple chromosomes or contigs could be enabled, to take advantage of the multicore infrastructure of modern PC.

After the batch run, results are combined and returned together with input data plus model parameters as a* biomvRCNS* class object, for which a plot method has been implemented to provide integrative visualization of the segmentation results with optional annotation.

### 2.3. Performance Comparison with Other Segmentation Methods

To show the reliability and relative performance of the proposed model, we compared our implementation with several other state-of-the-art segmentation algorithms (Table 1), using a similar approach as in [38], by calculating the receiver operating characteristic (ROC) curves on simulated data.

Some of the models reviewed in [40] have evolved over the years. Venkatraman and Olshen [14] present a faster, modified version of circular binary segmentation (CBS) [11] in R/Bioconductor package* DNAcopy*. Picard et al. [17] extend the univariate dynamic programming procedure [12] to joint analysis of multiple CGH profiles in R package* cghseg* and adopt the modified Bayesian information criterion [41] for model selection. We also included the unsupervised hidden Markov model described in R package* aCGH* [9] (labeled* HMM* hereafter) and the local adaptive weights smoothing procedure in R package* GLAD* [10] in our comparison; these are considered to be early efforts in the field, thus they can serve as baselines to show advances in the approaches.

In recent years, several new methods and computational tools have also been introduced. In R package* bcp* [16], Erdman and Emerson implement an efficient Bayesian change point model described by Barry and Hartigan [42]. Ben-Yaacov and Eldar suggest an ultrafast segmentation model based on wavelet decomposition and thresholding in R package* HaarSeg *[15]. Marioni et al. implement a heterogeneous hidden Markov model* bioHMM* [13] in R package* snapCGH*, which can utilize positional information or clone quality in the modeling process and, thus, could be considered as an extension of the* HMM* in package* aCGH*. Among these models, there has been no comparison study between* bcp*,* bioHMM*, and* HaarSeg* in recent literature. We did not include implementations that are specific to SNP data in our comparison, mainly due to the unique nature of the platform, which is less general in terms of segmentation and may require more inputs like B Allele Frequency (BAF) or genotype call, in addition to the copy number profile in the form of Log R Ratio (LRR).

### 2.4. Data Simulation for Algorithm Comparison

For the data simulation, we attempt to make it conceptually similar to the scenario one may encounter in real experiments. For copy number studies using CGH or using sequencing with matched case-control sample, three states are commonly assumed, and regions of copy gain and loss are of major interest when sizes range from about 1 kb to several megabases [43]. For this purpose, we first create pools of segments for each state; lengths of the segments are sampled from three Poisson distributions, with lambda equal to 20, 270, and 10, respectively. The distance between data points is assumed to be regular and equal to 1. Signal intensities are sampled from three normal distributions, *N*_1_(*r*, 1), *N*_2_(2 × *r*, 1), and *N*_3_(3 × *r*, 1) for each state, respectively, with state mean controlled via a ratio factor *r* varying from 1 to 3 at a step of 1. Segments from different states are then randomly sampled and joined together to form one data sequence.

For sequencing data, to check for splicing and novel transcripts or detect peaks for transcript factor binding sites, one would be interested in distinguishing the true expression signal from the background. Normally, annotated coding or noncoding transcripts are relatively much shorter compared to intergenic regions. In this case, we also first create pools of segments for three virtual states, intergenic, short, and relatively lowly-expressed gene and protein coding sequence with high abundance; lengths of the segments are sampled from three Poisson distributions, with lambda equal to 285, 5, and 10, respectively. Signal intensities for each segment are then sampled from three pools of Poisson distribution, *P*_1_(1), *P*_2_(*r*), and *P*_3_(*r* ^2^), with mean controlled via a ratio parameter *r* varying from 1.5 to 2 at a step of 0.25 for each pool of segments. Segments from different states are then randomly sampled and joined together to form one data sequence, representing one targeted region.

### 2.5. Performance Comparison Using ROC Curves

In this work, we compare our model with several well-tested segmentation algorithms, all of which are available as R packages. Since different algorithms tend to be tuned differently to suit their own methodologies for better sensitivity, here we do not attempt to alter their default settings and feed only the simulated signals without other information to the models, thus achieving an essentially fair comparison and mimicking a common-use case for normal users.

We use simulated data with varying levels of interstate ratio *r*, which is conceptually similar to signal-to-noise ratio (SNR); since, for both simulations, states with extreme values are of interest, the differences in mean between the extreme states and the intermediate states could be considered as signal, while the variation associated with the intermediate state could be considered as noise. We calculate the true-positive rates (TPR) and the false-positive rates (FPR) over 10000 iterations (100 simulations for each of the 100 random segments formation) of simulation for each level of *r*.

The TPR is defined as the number of points that are from the states of interest and fall into the predicted states of interest, divided by the total number of points from the states of interest. The FPR is defined as the number of points that are not from the states of interest but fall into the predicted states of interest, divided by the total number of points not from the states of interest. The true states of interest depend on the type of simulation; for normal data in simulation 1, this is assigned to the first and the third states, namely the gain and loss states, respectively. For count data in simulation 2, this is assigned to the third state, which is used to represent signal peak. The prediction is done by comparing the estimated segment mean with a threshold (*t*) varying from the maximum to the minimum of the simulated value. For abnormal state of gain in simulation 1 and peak in simulation 2, the segment with estimated value above the threshold is considered as positive; for state of loss in simulation 1, the segment with estimated value below the threshold is considered as positive. Definitions of TPR and FPR are formulated as follows:

(2) TPRloss=N(x<t ∣ s=1)N(s=1),FPRloss=N(x<t ∣ s≠1)N(s≠1),TPRgain ∣ s2=N(x>t ∣ s=3)N(s=3),FPRgain ∣ s2=N(x>t ∣ s≠3)N(s≠3).

All calculations were carried out in the statistical language R (version 3.0.1). Area under the curve (AUC) was estimated using Bioconductor package ROC (version 1.36.0). The system used for benchmarking is a standard 64 bits Linux desktop with Intel Core i7 with 3.07 GHz and 6 GB DDR3 memory.

## 3. Results

### 3.1. Performance Comparison with Simulated Data

**Figure 1:**
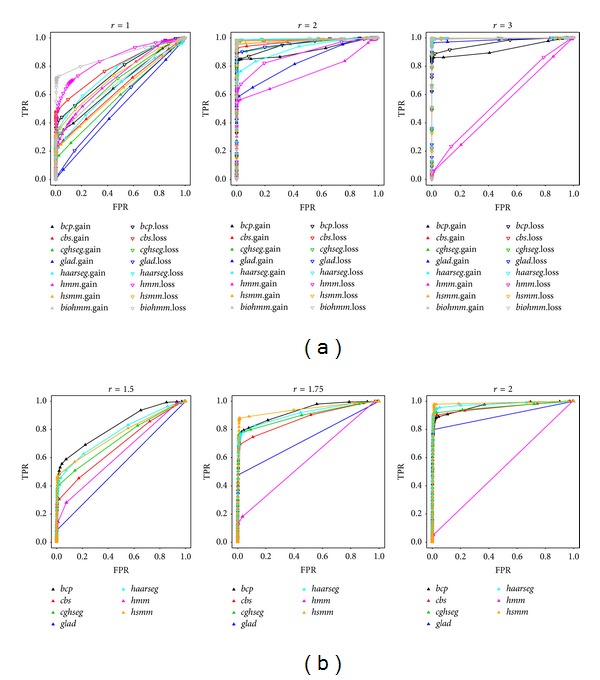
ROC curves for performance comparison. Receiver operating characteristic (ROC) curves for segmentation algorithm comparison under different signal-to-noise settings (*r*). Curves were generated by measuring the sensitivity and specificity at different threshold levels. The *x*-axis and *y*-axis show the false-positive rate (FPR) and true-positive rate (TPR), respectively. The upper panel (a) shows simulation 1, similar to an aCGH analysis, and the lower panel shows simulation 2, similar to peak identification using NGS. Compared algorithms are color-coded as indicated in the figure legend, while the up-triangle represents segment of gain in simulation 1 and peak in simulation 2, and hollow down-triangle represents segment of loss in simulation 1. Models are labeled using lowercase letters of their name. Our proposed model is coded as “*hsmm*” for simplicity and the hidden Markov model in package* aCGH* is labeled as “*hmm.*”

**Figure 2:**
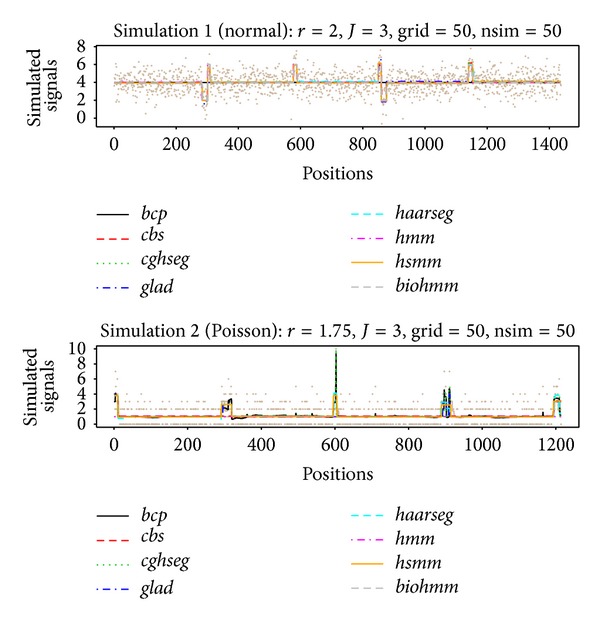
Examples of stimulated data and estimated segments. Two sets of randomly simulated data (chosen from the 50th random grid formation and the 50th iteration of that formation), one for each simulation run (using the intermediate *r* level, 2 for simulation 1 and 1.75 for simulation 2), are illustrated as an example with estimated segments from competing models. Segments are represented using the estimated segment averages. The true underlying grid used for data simulation is shown as the solid line in beige.

After two extensive simulation runs, we show the resulting ROC curves under different signal-to-noise ratios for all compared models in Figure 1. In Figure 2, two sets of randomly simulated data (chosen from the 50th random grid formation and the 50th iteration of that formation), one from each simulation run (using the intermediate *r* level, 2 for simulation 1 and 1.75 for simulation 2), have been illustrated as an example together with estimated segments from competing models.

In simulation 1 (Table 2), most algorithms—except for* HMM*—perform comparably well at intermediate- and low-noise scenarios. The difference in detecting gain and loss is consistent with our simulation setup, where we intentionally set the loss region to be relatively longer, making it easier to detect. In general, the competing algorithms can be categorized into three performance groups: our model,* bioHMM*, and* HaarSeg* perform best, followed by* CBS* and* cghseg*, and the last three algorithms perform less satisfactorily. Notably, in simulation 1,* bioHMM* has surprisingly high power in a high-noise setting. However, the advantage essentially disappears when signals get stronger. This phenomenon could result from its model selection process, where it attempts to assign a higher number of states, thus more segments, to compensate for the random noise. Additionally,* HaarSeg* has difficulty detecting short gain segments, which could be related to the default model setting that is not well adapted to short aberrations [15].

A smoothing algorithm like* GLAD* only operates well under higher signal-to-noise ratio. Smoothing results in less accurate segment boundaries. Further, as mentioned in [40],* GLAD* is sensitive to single outliers, which explains the minor deficiency of sensitivity in detecting gain regions even for low-noise cases. The behavior of* bcp* indicates that to achieve higher power specificity must be lost, even with a high signal-to-noise setting.* HMM* achieves a high area under the curve (AUC) when high noise exists (*r* = 1) in simulation 1 and performs comparably worse when signals are stronger, eventually failing to identify most segments. This is in accordance with [40], where* HMM* failed to identify any region in Glioblastoma Multiforme (GBM) data. It also fails to make any meaningful segmentation in simulation 2.

In simulation 2 (Table 3), when data contain a mixture of Poisson distributions, we failed to run* bioHMM* due to an error in a foreign function call to the C library. We have to assume that the implementation cannot work on discrete count data. However, all other implementations are still operable and achieve similar performance as in simulation 1. Though the mean parameter for Poisson data simulation is not considerably large, the normal approximation could still achieve reasonably good power. Nonetheless, our explicit modeling of count data remains advantageous for segmenting count data, which has the highest weighted average rank (Table 4), followed by* bcp* and* HaarSeg*. Compared to* HaarSeg*, the power boost for* bcp* essentially occurs under higher FPR. It is possible that algorithms like* bcp* perform better when a stronger signal exists, which could be due to the normal error assumption in the model.

For both simulations we could confirm that, as has been shown in [40],* cghseg* and* CBS* perform consistently well under various scenarios. Two of the newly introduced methods,* bioHMM* and* HaarSeg*, also exhibit comparable or better performance; in contrast, our model consistently ranks among the top 3 performing algorithms. Across the two simulations,* HMM* and* GLAD *are considered to possess lowest power, while for* bcp* an associated high error rate is observed.

Concerning computation time,* HaarSeg* is the fastest algorithm among all implementations, by a factor of 50–100;* bioHMM* is the slowest due to its internal model selection process.* bcp* is the second slowest, as a result of long Markov chain Monte Carlo (MCMC) run. The processing time of our model is similar to* cghseg* and is slower than* CBS*, which is about two times faster.

We also took a closer look at the overall accuracy of estimated segment number, in Table 4. For both simulations, we joined, on average, 14 segments into one sequence. Occam's razor states that the best model should be the simplest yet still retain the same power. In simulation 1, our model achieves the lowest rooted mean-squared error (RMSE) and mean absolute error (MAE); in simulation 2, our model finds fewer segments: the median number of detected segments was only 6 across three noise levels. Taking into account the power advantage of our method in the performance comparison, this finding indicates that the estimated segment boundaries are more accurate in our model. Simulated aberrant segments are sampled from the same distribution, and the sojourn modeling in our method takes advantage of this property. In the second simulation,* HaarSeg* achieves the lowest error estimates for both RMSE and MAE.* cghseg* gives error estimates similar to our model. For* HaarSeg* and* cghseg*, this is essentially achieved by fitting more segments. As has been pointed out in [12], assumptions of the mean-variance relationship imposed on the model may lead to more segments to satisfy such requirements.

## 4. Example of Differentially Methylated Region (DMR) Detection

Differentially methylated regions (DMRs) are genomic regions with different methylation status, that is, variable degrees of DNA methylation between different samples, which has been considered to have regulatory functions for gene transcription [44] and is associated with cell differentiation and proliferation [45, 46]. Such regions can be surveyed using high-throughput technologies like tiling array [47] and sequencing [48].

As an example, we include a set of data extracted from* BiSeq* [49], which contains a small subset of a published study [50], comprising intermediate differential methylation results before DMR detection. We first load the “variosm” data,

 > *library (biomvRCNS)*
 > *data (variosm)*

The data contains a* GRanges* object* variosm* with two meta columns: “*meth.diff*,” methylation difference between the two sample groups and “*p.val*,” significance level from the Wald test. Our model could be applied on data from other pipelines as well, using similar data input.

In the* BiSeq* workflow, they use an approach similar to the max-gap-min-run algorithm to define DMR boundaries, by prior filtering and comparing the differential test statistics with a user-specified significance level in the candidate regions. The positional information for methylation sites is accounted for by locating and testing highly correlated cluster regions in the filtering process.

With* biomvRhsmm*, we utilize both types of information to detect DMRs: (1) the difference in the methylation ratio and (2) the significance level from the differential test. The methylation difference gives information about the directionality of the change as well as the size, and the significance level gives the confidence in claiming differential events. We implicitly ask the model to give 3 states, since *J* is default to 3. Regarding the methylation ratio “*meth.diff*,” these levels may be hypomethylated regions, undefined null regions, or hypermethylated regions, respectively. When modeling significance levels “*p.val*,” these states would represent high confidence regions, low confidence regions, or null results. For both scenarios, we are more interested in extreme states, where we have consistent direction of differences and low* P* values. However, the distributions of observation values in “*meth.diff*” and “*p.val*” are both nonuniform and asymmetric around 0 (for “*meth.diff*”) and 0.5 (for “*p.val*”), thus we enable the cluster mode for emission prior to initialization by setting* prior.m=*“*cluster*.” The “*cluster*” mode will employ the method described in [51] to divide data into clusters and then use the centroid of each cluster to represent the mean parameter; further, the variance structure or other distributional parameters can be estimated using the corresponding clusters.

Due to the nonuniformly located CpG sites, one may split inter-spreading long segments with parameter max gap = 100 (see code chunk in Box 1).

**Figure 3:**
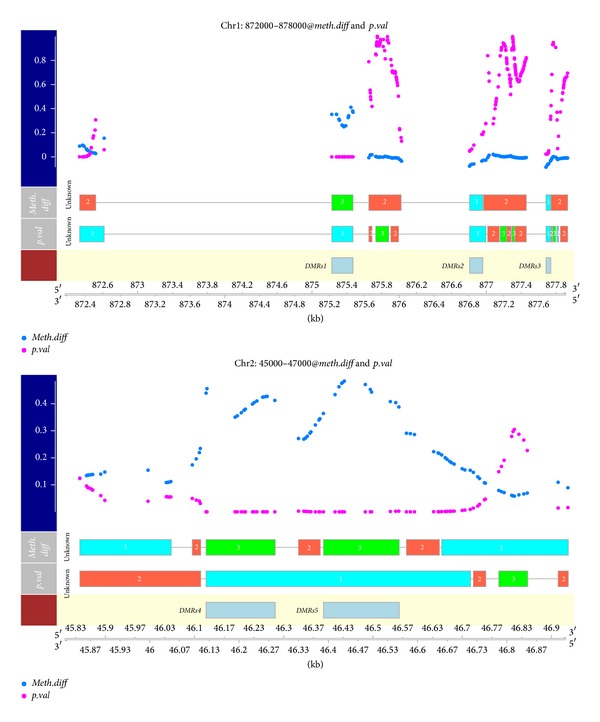
Detected differentially methylated regions (DMRs) in the example data, together with estimated segmentation profiles. DMRs could be located by intersecting resulting states “1” or “3” in “*meth.diff*” and segment “1” in “*p.val*,” like has been shown in the code chunk, indicated by boxes in the third row.

After the model fitting, by intersecting regions with extreme “*meth.diff*” and regions with low “*p.val*,” we can locate those detected DMRs, returned with their average “*meth.diff*” and “*p.val*”. Compared to the regions detected in the* BiSeq* vignette, the two sets of regions are largely similar except for two regions. First chr1: 872335, 872386 had highly asymmetric distribution of “*meth.diff*.” Another region, chr2: 46915, 46937, resides in the tail of chromosome 2 and has only 2 methylation sites; this was sorted into the intermediate state due to the lack of support from both the emission level and the sojourn time. However, due to the filtering applied in* BiSeq* workflow, they built wider regions out of a smaller set of more significant sites; in contrast, our approach has more refined regions, and we identified two hypomethylated regions (chr1: 876807, 876958 and chr1: 877684, 877738). The two segmented profiles are depicted in Figure 3, using the default plot method.

## 5. Discussion

The segmentation problem, in general, occurs in many types of biological experiments and can naturally fit into the hidden Markov model framework with segment boundaries modeled as transitions between hidden states.

As a generalization of the hidden Markov model, HSMM allows the sojourn distribution to be specified other than the Geometric distribution implicitly used in common HMM. Given the complexity of the genome, such an implicit assumption could be easily violated. Though the true underlying sojourn distributions involving various genomic features remains unknown, our implementation gives more flexible options in the modeling and, thus, might provide more insight.

In this package, several types of sojourn distribution are implemented. For example, with gamma-distributed sojourn, the neighboring position will tend to stay in the same state and transit to other states if far apart. Differing from the original design in [38], our implementation utilizes the positional information naturally associated with most genomic features for the sojourn density estimation. Such an integrative approach is advantageous over simply using the rank of feature positions, since mapping positions are not always uniformly distributed and the spatial patterns may be of interest in experiments like DMR detection. Further, HSMM differs from those models that embed positions in a nonparametric fashion, like* BioHMM* [13] and QuantiSNP [19], or as in the “instability-selection” model for LOH analysis [22, 23]; these all employ variations of exponential function to account for feature position. Our HSMM is closer to the DBN model employed in Segway [34] but is less experiment-specific and easier to interpret and has convenient communication with other analytical and visualization tools within the Bioconductor community.

The explosion of data availability provides another possibility of learning from previous studies. Other than the flat prior commonly used in Bayesian inference, prior information for the sojourn density could be estimated from annotation or previous studies, thus it can be effectively utilized together with positional information of features to guide the estimation of the most likely state sequence.

With its full probabilistic model, various emission densities are provided, enabling the model to handle normally distributed data from traditional array platforms as well as counting data from sequencing experiments. The proposed model has also been applied on a well-studied aCGH dataset from Coriell cell lines [7] and from RNA-seq data generated by the ENCODE project [52, 53] to illustrate its other functionalities in the package vignette.

## 6. Conclusions

In this work, we present a novel hidden semi-Markov model designed specifically for genomic data analysis. The proposed model has been compared with several other state-of-the-art segmentation methods; our implementation is efficient and achieves comparable or better sensitivity and specificity in genomic segmentation. Further, our model has flexible data distribution assumption, enabling a unified interface for segmenting data generated from different experimental platforms. By incorporating genomic positions into the sojourn distribution of HSMM, with optional prior learning using annotation or previous studies, model output is more biologically sensible. To this end we would like to present our model as a general segmentation engine to serve in a wide range of genomic research.

## 7. Availability and Requirements

Function* biomvRhsmm* is implemented as part of the R/Bioconductor package* biomvRCNS*, which is available from the Bioconductor project.

 Function name:* biomvRhsmm*
 Package name:* biomvRCNS*
 Project home page:
 
http://bioconductor.org/packages/devel/bioc/html/biomvRCNS.html
 Operating system(s): Linux, Mac OS X, Windows
 Programming language: R, C
 Other requirements: R (≥ 3.0.0)
 License: GPL (≥2).

## Supplementary Material

Further mathematical details of the forward-backward EM procedure and the Viterbi algorithm implemented in the proposed hidden semi-Markov model.

## Figures and Tables

**Box 1:**
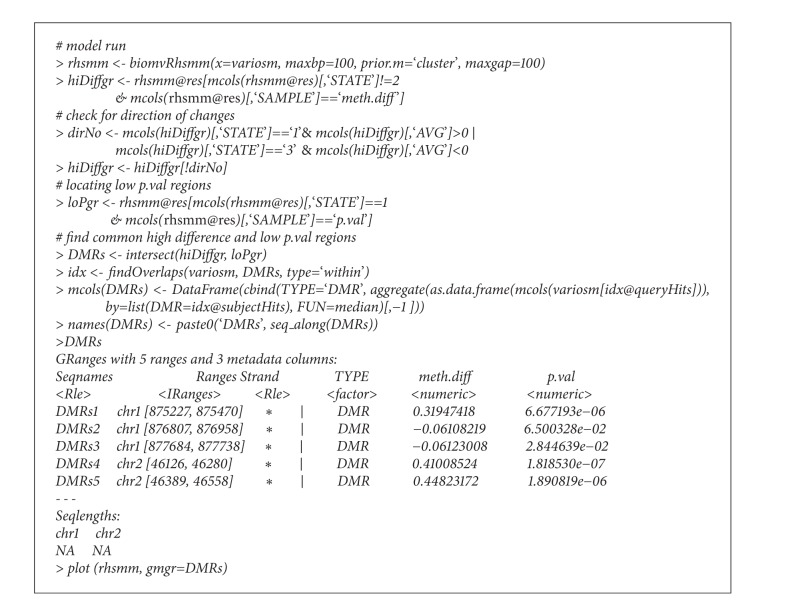
Code chunk.

**Table 1:** List of algorithms compared in this paper.

Name	Reference	Method	R package (version)
*bcp *	Erdman and Emerson (2008) [16]	Product Partition Model	bcp_3.0.1
*bioHMM *	Marioni et al. (2006) [13]	Heterogeneous HMM	snapCGH_1.30.0
*CBS *	Venkatraman and Olshen (2007) [14]	Modified Circular Binary Segmentation	DNAcopy_1.34.0
*cghseg *	Picard et al. (2011) [17]	Joint CGH Segmentation	cghseg_1.0.1
*GLAD *	Hupé et al. (2004) [10]	Adaptive Weights Smoothing	GLAD_2.24.0
*HaarSeg *	Ben-Yaacov and Eldar (2008) [15]	Wavelet Decomposition and Thresholding	HaarSeg_0.0.3
*HMM *	Fridlyand et al. (2004) [9]	Homogeneous HMM	aCGH_1.38.0

**Table 2:** Area under the ROC curves of simulation 1 data.

	*r* = 1		*r* = 2		*r* = 3		Weighted avg. rank
	AUC_g_	AUC_l_	AUC_g_	AUC_l_	AUC_g_	AUC_l_	
*hsmm *	0.619487	0.729668	0.982176	0.988689	0.999141	0.998519	3.528400
*bcp *	0.675283	0.758042	0.912009	0.956031	0.921090	0.963401	6.416511
*bioHMM *	0.685575	0.875202	0.977079	0.990215	0.995062	0.997551	3.408419
*CBS *	0.633272	0.795941	0.974008	0.985508	0.996065	0.995409	4.491084
*CGHseg *	0.586505	0.696329	0.960183	0.991765	0.996045	0.998051	4.621081
*GLAD *	0.506588	0.548763	0.833011	0.962862	0.986098	0.996577	6.907573
*HaarSeg *	0.649687	0.763682	0.923416	0.993653	0.995908	0.998408	3.886978
*HMM *	0.717595	0.854783	0.749492	0.887209	0.526358	0.573611	6.589848

AUC_g_ and AUC_l_ are area under the receiver operating characteristic (ROC) curves for simulated gain and loss segments, respectively, for each *r*.

Weighted avg. rank is calculated as *n* + 1 − ∑_*j*=1_^*j*=*c*^AUC_*i*_ × rank^*j*^(AUC_*i*_)/*c* for each model *i*, where *c* is the number of AUC columns and *n* is the number of competing models.

**Table 3:** Area under the ROC curves of simulation 2 data.

	AUC_*r*=1.5_	AUC_*r*=1.75_	AUC_*r*=2_	Weighted avg. rank
*hsmm *	0.7623442	0.9423881	0.9849165	2.232382
*bcp *	0.8219388	0.9280808	0.9656302	2.616598
*CBS *	0.6828102	0.874411	0.954873	5.487906
*CGHseg *	0.7292456	0.8982693	0.9594827	4.550670
*GLAD *	0.5418485	0.7366826	0.8971196	6.730182
*HaarSeg *	0.7728192	0.9114189	0.9786983	2.977933
*HMM *	0.6243222	0.5872855	0.5259849	7.212695

Weighted avg. rank is calculated as *n* + 1 − ∑_*j*=1_^*j*=*c*^AUC_*i*_ × rank^*j*^(AUC_*i*_)/*c* for each model *i*, where *c* is the number of AUC columns and *n* is the number of competing models.

**Table 4:** Processing time and error estimate of the compared models.

	avg.*t*	Simulation 1			Simulation 2		
		avg.cp	MAE	RMSE	avg.cp	MAE	RMSE
*hsmm *	0.25645	12/11.16	5.756	3.476	6/6.50	18.73	6.31
*bcp *	1.46298	NA	NA	NA	NA	NA	NA
*bioHMM *	6.96811	14/14.71	8.655	7.376	NA	NA	NA
*CBS *	0.12168	12/11.02	7.444	4.178	5/4.93	20.762	7.068
*CGHseg *	0.28938	12/9.89	9.059	4.783	7/7.00	14.83	5.533
*GLAD *	0.23725	10/8.22	13.128	6.071	3/4.15	22.139	7.668
*HaarSeg *	0.00268	17/15.57	12.896	4.984	10/10.65	10.117	4.018
*HMM *	0.28008	7/38.97	94.666	80.792	1/60.05	144.83	97.178

avg.*t* is calculated as the mean run time of 20000 simulation iterations.

avg.cp is the median/mean number of segments estimated across 3 SNR settings.

MAE is calculated as the mean absolute error ∑|no.seg − true.no.seg|/*n*.

RMSE is the rooted mean squared error $\sqrt{\sum {\left(\text{no.seg}-\text{true.no.seg}\right)}^{2}/n}$.

NA indicates that the measurement is not applicable for this algorithm. For *bcp*, the model output posterior means for each position that does not tend to form segments with constant mean. For *bioHMM*, the model cannot be run, thus no results were collected.
