# Supplementary material for: biomvRhsmm: Genomic Segmentation with Hidden Semi-Markov Model
Source: Biomed Res Int. 2014 Jun 3;2014:910390. doi: 10.1155/2014/910390 (PMC4065698; doi:10.1155/2014/910390)
Supplement: Supplementary file 1 — Further mathematical details of the forward-backward EM procedure and the Viterbi algorithm implemented in the proposed hidden semi-Markov model. [file 910390.f1.pdf]

Supplementary material for: " biomvRhsmm: Genomic segmentation with hidden semi-Markov mode"

Yang Du, Eduard Murani, Siriluck Ponsuksili and Klaus Wimmers

February 20, 2014

1 Hidden semi-Markov model definition

To start with, let's make a brief summary of the concepts involved and introduce the hidden semi-Markov model formulation. For some experimental data X, we have a vector of observations $x_t = (x_t^1, \ldots, x_t^N)$ made for N samples at each time or position t, $t = 1, \ldots, T$. At each t, there is an underlying unobserved state $S_t \in S = \{1, \ldots, J\}$, which depends only on the previous state at $t - 1$, thus forming a length T discrete Markov chain with a finite number J possible states. The initial state probability is determined by distribution π, $\pi_j = P(S_1 = j)$, $j = 1, \ldots, J$, with $\sum_{j=1}^{J} \pi_j = 1$ and $\pi_j \geq 0$. The conditional probability distribution of the observed variable x_t given the unobserved (or hidden) state J, $b_j(x_t) = P(X_t = x_t \mid S_t = j)$, is controlled via the emission probability distribution B. The transition probability distribution A, governing the probability of moving from one state to another, is formulated as $a_{ij} = P(S_{t+1} = j \mid S_t = i)$, with $\sum_{j=1}^{J} a_{ij} = 1$ and $a_{ij} \geq 0$. Thus a HMM can be defined by $\theta = (\pi, A, B)$, for which a schematic of the model parametrization is shown in Fig. 1.

A semi-Markov chain could be considered as a two-layer mixture, an embedded first-order Markov chain representing the transitions between distinct states, which follows the standard definition of HMM, and an occupancy distribution attached to each non-absorbing state of the embedded first-oder Markov chain.

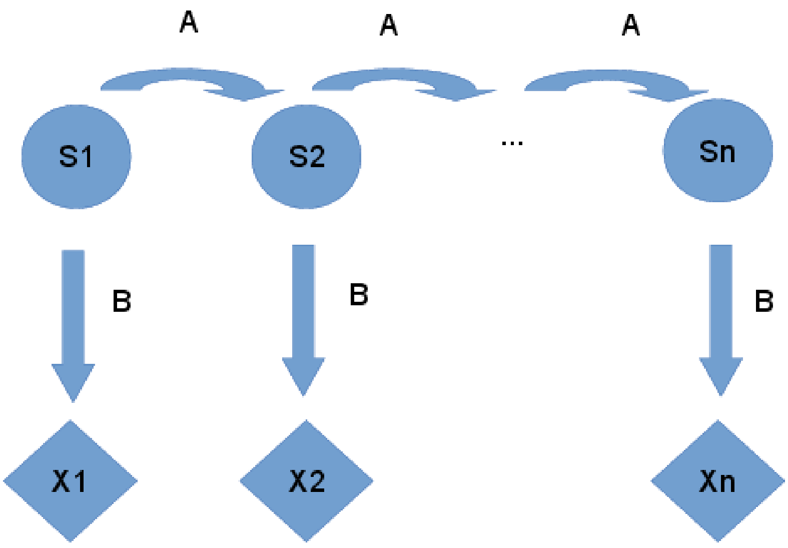

Figure 1: Schematic of HMM parametrization

The discrete state occupancy distribution or the sojourn distribution, D, is defined as the probability of spending u consecutive time steps in state j,

$$d_j(u) = P(S_{t+u+1} \neq j, S_{t+u-v} = j, v = 0, \dots, u-2 \mid S_{t+1} = j, S_t \neq j), \quad u = 1, \dots, M_j \tag{1}$$

where M_j denotes the upper bound to the time spent in state j. For a normal HMM, the sojourn time could be simply deducted to $d_j(u) = a_{jj}^{u-1}(1 - a_{jj})$, which is geometrically distributed. HSMM, with the sojourn distribution explicitly specified using a common distribution or non-parametrically estimated using a pseudo sample, could be defined by $\theta = (\pi, A, B, D)$. A complete likelihood of the HSMM is given in Guédon [2003] with survivor function $D_j(u) = \sum_{v \geq u} d_j(v)$ representing the sojourn time spent in the

last state and number of distinct states$R$

$$
\begin{aligned}
L(\theta) = \pi_{S_1} d_{S_1}(u_1) \left\{ \prod_{r=2}^{R} P(S_r \mid S_{r-1}) d_{S_r}(u_r) \right\} \\
\cdot P(S_R \mid S_{R-1}) D_{S_R}(u_R) \prod_{t-1}^{T} P(X_t \mid S_t)
\end{aligned}
\tag{2}
$$

where S_r is the rth state visited in the first-order Markov chain transition and S_R is the last states visited.

With likelihood function defined (Eq. (2)), the optimal model parameters θ could then be estimated using the EM algorithm. A forward-backward algorithm for the estimation step and a Viterbi algorithm to derive the most likely state sequence are explained in Guédon [2003], where the author also shows the possibility of replacing the non-parametric M-step of the EM algorithm in sojourn distribution parameters re-estimation with a parametric M-step in practice, to simplify model and prevent over-fitting.

2 Estimation of hidden semi-Markov model

For the estimation step, as have been illustrated in Guédon [2003], the forward recursion is first given by,

$$\begin{aligned} F_j(t) &= P(S_{t+1} \neq j, S_t = j \mid X_1^t = x_1^t) \\ &= \frac{b_j(x_t)}{N_t} \left[\sum_{u=1}^{t} \left\{ \prod_{v=1}^{u-1} \frac{b_j(x_{t-v})}{N_{t-v}} \right\} d_j(u) \sum_{i \neq j} a_{ij} F_i(t-u) \right. \\ &\quad \left. + \left\{ \prod_{v=1}^{t} \frac{b_j(x_{t-v})}{N_{t-v}} \right\} d_j(t+1) \pi_j \right], \end{aligned} \tag{3}$$

where $t = 1, \ldots, T-1$, $j = 1, \ldots, J$, and N_t is the normalizing factor, which could be derived during the forward recursion using Eq. (4). $X_1^t = x_1^t$ is the shorthand form of $(X_1 = x_1, X_2 = x_2, \ldots, X_t = x_t)$, the same analogous abbreviation is also used for $S_1^t = s_1^t$. For the last state visited when $t = T$, the exact duration of the stay is unknown, however using the minimal staying time, the sojourn density $d_j(u)$ could be replaced by the survivor function $D_j(u)$.

$$\begin{aligned} N_t &= P(X_t = x_t \mid X_1^{t-1} = x_1^{t-1}) \\ &= \sum_j b_j(x_t) \left[\sum_{u=1}^{t} \left\{ \prod_{v=1}^{u-1} \frac{b_j(x_{t-v})}{N_{t-v}} \right\} D_j(u) \sum_{i \neq j} a_{ij} F_i(t-u) \right. \\ &\quad \left. + \left\{ \prod_{v=1}^{t} \frac{b_j(x_{t-v})}{N_{t-v}} \right\} D_j(t+1) \pi_j \right], \end{aligned} \tag{4}$$

The smoothed probability $L_j(t) = P(S_t = j \mid X_1^t = x_1^t)$ at each position for a hidden semi-Markov chain can be decomposed and written as,

$$\begin{aligned} L_j(t) &= P(S_t = j \mid X_1^t = x_1^t) \\ &= L1_j(t) + L_j(t+1) - P(S_{t+1} = j, S_t \neq j \mid X_1^T = x_1^T), \end{aligned} \tag{5}$$

where $L1_j(t) = P(S_{t+1} \neq j, S_t = j \mid X_1^T = x_1^T) = B_j(t) F_j(t)$ gives the conditional independence between future and past at transition between distinct states, which also provides the entry point for the backward recursion. $L_j(T)$ is initialized as $L_j(T) = P(S_T = j \mid X_1^T = x_1^T) = F_j(T)$ for $t = T$ and all j.

The backward recursion is done by pre-calculating another auxiliary variable, $G_j(t+1)$, which helps reduce the complexities of the forward-backward procedure to $O(JT(J+T))$ time and $O(JT)$ space in the worst case. $L1_j(t)$ and the third term in Eq. (5) could then be written as,

$$L1_j(t) = \left\{ \sum_{k \neq j} G_k(t+1) a_{jk} \right\} F_j(t), \tag{6}$$

$$P(S_{t+1} = j, S_t \neq j \mid X_1^T = x_1^T) = G_j(t+1) \sum_{i \neq j} a_{ij} F_i(t), \tag{7}$$

where $G_j(t+1) = \sum_{u=1}^{T-t} G_j(t=1,u)$, and

$$\begin{aligned} G_j(t+1,u) &= \frac{L1_j(t+u)}{F_j(t+u)} \left\{ \prod_{v=0}^{u-1} \frac{b_j(x_{t+u-v})}{N_{t+u-v}} \right\} d_j(u), u = 1, \ldots, T-1-t, \\ G_j(t+1,T-t) &= \left\{ \prod_{v=0}^{T-1-t} \frac{b_j(x_{T-v})}{N_{T-v}} \right\} D_j(T-t). \end{aligned} \tag{8}$$

For the parameter re-estimation step, the initial probabilities and transition probabilities could be updated at each EM iteration,

$$\hat{\pi}_j = P(S_1 = j | X_1^T = x_1^T; \theta) = L_j(1), \tag{9}$$

$$\hat{a_{ij}} = \frac{\sum_{t=1}^{T-1} G_j(t+1) a_{ij} F_i(t)}{\sum_{t=1}^{T-1} L1_i(t)}. \tag{10}$$

Using components calculated during the forward-backward run, the updates of state occupancy probabilities and emission probabilities are done as the following, Eq. (11), depending on the assumptions imposed on the emission distribution ($I(x_t)$) and sojourn distribution. Eq. (12) gives the non-parametric E-step as shown in [Guédon 2003].

$$\hat{b}_j(x_t) = \frac{\sum_{t=1}^{T} L_j(t) I(x_t)}{\sum_{t=1}^{T} L_j(t)}, \tag{11}$$

$$\hat{d}_j(u) = \frac{\eta_{ju}}{\sum_{t=0}^{T-1} L1_j(t) + L_j(T)}, \tag{12}$$

where the quantities η_{ju} could be computed during the backward procedure as in Eq. (13).

$$\begin{aligned}\eta_{ju} = &\sum_{t=1}^{T-1} P(S_{t+u+1 \neq j, S_{t+u-v}=j, v=0,\ldots,u-1}, S_t \neq j \mid X_1^T = x_1^T; \theta) \\ &+ P(S_u \neq j, S_{u-v} = j, v = 1, \ldots, u | X_1^T = x_1^T; \theta)\end{aligned} \tag{13}$$

The first term in Eq. (13) could be further re-written, when $u \leq T - 1 - t$,

$$P(S_{t+u+1 \neq j, S_{t+u-v}=j, v=0,\ldots,u-1}, S_t \neq j \mid X_1^T = x_1^T; \theta) = G_j(t+1, u) \sum_{i \neq j} a_{ij} F_i(t), \tag{14}$$

and for $u > T - 1 - t$,

$$P(S_{t+u+1 \neq j, S_{t+u-v}=j, v=0,\ldots,u-1}, S_t \neq j \mid X_1^T = x_1^T; \theta) = \left\{ \prod_{v=0}^{T-1-t} \frac{b_j(x_{T-v})}{N_{T-v}} \right\} d_j(u) \sum_{i \neq j} a_{ij} F_i(t) \tag{15}$$

The second term in Eq. (13) could also be represented using pre-computed products, when $u \leq T$,

$$P(S_u \neq j, S_{u-v} = j, v = 1, \ldots, u | X_1^T = x_1^T; \theta) = \frac{L1_j(u-1)}{F_j(u-1)} \left\{ \prod_{v=1}^{u} \frac{b_j(x_{u-v})}{N_{u-v}} \right\} d_j(u) \pi_j, \tag{16}$$

and for $u > T$,

$$P(S_u \neq j, S_{u-v} = j, v = 1, \ldots, u | X_1^T = x_1^T; \theta) = \left\{ \prod_{v=1}^{T} \frac{b_j(x_{T-v})}{N_{T-v}} \right\} d_j(u) \pi_j. \tag{17}$$

The quantities η_{ju} can also be treated as a pseudo-sample of some selected parametric sojourn distributions. Thus other parametric re-estimations basing on continuous and discrete distributions like Gamma, Poisson, and Negative Binomial distribution can be done *ad hoc*, using point estimation methods like moment estimator or maximum likelihood estimator, with additional shift parameter d to control the minimum stay duration in a state. The shift parameter d is determined by assessing possible values, $1, \ldots, min(u \mid \eta_{ju} > 0)$, of which gives the maximum likelihood of the re-estimated sojourn mass.

To obtain the most likely state sequence, a Viterbi procedure, using a similar forward recursion, could be applied by defining the quantities $\alpha_j(t)$ as maximum conditional likelihood of having a transition of state after the current t, $\alpha_j(t) = \max_{S_1,\ldots,S_t} P(S_{t+1} \neq j, S_t = j, S_1^{t-1} = s_1^{t-1}, X_1^t = x_1^t)$. For $t \leq T-1$, $\alpha_j(t)$ could be re-written as,

$$\begin{aligned}
\alpha_j(t) = & \max_{S_1,\ldots,S_{t-1}} P(S_{t+1} \neq j, S_t = j, S_1^{t-1} = s_1^{t-1}, X_1^t = x_1^t) \\
= & b_j(x_t) \max \left[\left\{ \prod_{v=1}^{t} b_j(x_{t-v}) \right\} d_j(t+1)\pi_j, \right. \\
& \left. \max_{1 \leq u \leq t} \left[\left\{ \prod_{v=1}^{u-1} b_j(xt-v) \right\} d_j(u) \max_{i \neq j} \{ p_{ij}\alpha_i(t-u) \} \right] \right].
\end{aligned} \tag{18}$$

While for $t = T$ again using $D_j(t)$, the right censoring of the sojourn time in the last state visited, $\alpha_j(T)$ is formulated as the following,

$$\begin{aligned}
\alpha_j(T) = & \max_{S_1,\ldots,S_{T-1}} P(S_T = j, S_{T-1} = j, S_1^{t-1} = s_1^{t-1}, X_1^T = x_1^T) \\
= & b_j(x_T) \max \left[\left\{ \prod_{v=1}^{T} b_j(x_{T-v}) \right\} D_j(T+1)\pi_j, \right. \\
& \left. \max_{1 \leq u \leq T} \left[\left\{ \prod_{v=1}^{u-1} b_j(xT-v) \right\} D_j(u) \max_{i \neq j} \{ p_{ij}\alpha_i(T-u) \} \right] \right].
\end{aligned} \tag{19}$$

Thus the most likely state sequence associated with the observed data sequence could then be backtracked by finding the j which maximize $\alpha_j(t)$.

The E-step of the forward-backward EM procedure and the Viterbi algorithm described in Guédon [2003] has been implemented as C library in the R/Bioconductor package *biomvRCNS*, serving as the core of our proposed hidden semi-Markov segmentation model.

References

Guédon, Y. (2003). Estimating Hidden Semi-Markov Chains from Discrete Sequences. *Journal of Computational and Graphical Statistics*, **12**(3), 604–639.
